# Radiological Improvement of Adolescent Idiopathic Scoliosis Following an Integrated Postural Reprogramming Approach: A Retrospective Case Series

**DOI:** 10.3390/diagnostics16040514

**Published:** 2026-02-09

**Authors:** Mirko Zisi, Sara Bizioli, Lorenzo Mosca, Francesco Tucci, Vincenzo Canali

**Affiliations:** 1 Habitus et Motus, Via Nazionale 144 Alto Reno Terme, 40046 Alto Reno Terme, Italy; 2 Postura e Sport Srl, Via Alberto Savinio 26F, 43123 Parma, Italyinfo@posturaesport.com (V.C.)

**Keywords:** adolescent idiopathic scoliosis, Cobb angle, radiological outcomes, conservative scoliosis treatment, postural reprogramming, spinal radiography, motor learning-based rehabilitation, orthopedic brace

## Abstract

**Background/Objectives**: Adolescent idiopathic scoliosis (AIS) is a three-dimensional spinal deformity commonly managed with conservative strategies, including bracing and physiotherapeutic scoliosis-specific exercises (PSSEs). The Canali Postural Method^®^ (CPM) is an individualized kinesiological approach aimed at postural reprogramming, while the Canali Orthopedic Brace is an intermittent, non-rigid device intended to facilitate active postural control rather than continuous passive correction. **Case Presentation**: We retrospectively report two adolescent females with thoracolumbar rotoscoliosis (Risser grade 4). Case 1 (15 years) presented with a left-convex thoracolumbar curve (apex T12–L1) with a Cobb angle of 19.4° and a derotation angle ratio (DAR) of 1.9. Case 2 (16 years) presented with a right-convex thoracolumbar curve (apex T10) with a Cobb angle of 41.14° and a DAR of 3.7. Both patients underwent supervised CPM-based exercise sessions combined with intermittent use of the Canali Orthopedic Brace. **Discussion and Conclusions**: Follow-up radiographs showed a marked reduction in curve magnitude and rotational parameters: in Case 1, the Cobb angle decreased from 19.4° to 4.1° and DAR from 1.9 to 0.4; in Case 2, the Cobb angle decreased from 41.14° to 15.17° and DAR from 3.7 to 1.36. Pelvic asymmetry was also reduced, and no worsening of sagittal alignment was observed. Given the retrospective design, the small sample size, heterogeneity in intervention duration, and the lack of clinical outcomes and formal measurement reliability testing, these findings should be interpreted with caution and warrant confirmation in prospective controlled studies.

## 1. Introduction

Adolescent idiopathic scoliosis (AIS) is a three-dimensional spinal deformity characterized by coronal deviation, axial rotation, and alterations of sagittal alignment [1]. Conservative management is commonly recommended for mild-to-moderate curves to reduce the risk of progression during growth and to limit the need for surgical correction. Standard conservative strategies include rigid or semi-rigid bracing and exercise-based interventions [2,3,4].

In recent years, physiotherapeutic scoliosis-specific exercises (PSSEs), such as Schroth-based programs and the Scientific Exercise Approach to Scoliosis (SEAS), have gained increasing attention. These approaches emphasize active self-correction, three-dimensional postural control, and motor learning principles, and are often used alone or in combination with bracing in selected patients [5,6].

The Canali Postural Method^®^ (CPM) [7] is an individualized kinesiological approach aimed at postural reprogramming through progressive exercises targeting sagittal stabilization, transverse plane control, and frontal plane asymmetry. In addition, the Canali Orthopedic Brace is a non-rigid device intended for intermittent use, with the proposed aim of facilitating postural awareness and active control rather than providing continuous passive correction.

Evidence supporting active, motor learning-based strategies combined with intermittent non-rigid orthotic use in AIS remains limited, particularly in adolescents with advanced skeletal maturity [8]. Therefore, the aim of this retrospective case series is to report radiological outcomes (Cobb angle and rotational parameters) in two adolescent patients with thoracolumbar AIS treated with an integrated approach combining CPM-based supervised exercises and intermittent use of the Canali Orthopedic Brace.

## 2. Materials and Methods

The patients were treated with an integrated conservative intervention combining individualized postural exercises based on the Canali Postural Method (CPM) and intermittent use of the Canali Orthopedic Brace. Intervention dose differed between cases: in Case 1, the program consisted of 30 supervised sessions delivered once weekly (approximately 30 min/session), whereas in Case 2, supervised sessions were delivered over one year at a frequency of twice weekly (approximately 60 min/session).

The exercise program was individualized following a clinical and postural assessment aimed at identifying asymmetries in neuromuscular dominance and compensatory movement patterns associated with the scoliotic posture. Particular attention was given to trunk stabilization capacity and the coordination between the pelvis and thoracic cage during postural control tasks.

Exercises were selected according to CPM principles and focused on improving neuromuscular control and reducing compensatory strategies during trunk alignment [9,10,11,12]. The intervention targeted sagittal plane stabilization, transverse plane (derotational) control, and frontal plane asymmetry, with exercises directed against the convexity of the thoracolumbar curve.

The exercise sequence included the following: (i) sagittal plane activation and stabilization to improve axial load distribution; (ii) exercises targeting rotational control; and (iii) frontal plane exercises aimed at reducing lateral asymmetry and dominant loading toward the convex side. Exercises were performed primarily under isometric or low-dynamic conditions to limit compensatory movements and promote controlled alignment. Progression was based on qualitative improvement in postural alignment and motor control rather than increases in intensity or volume [13,14].

Sessions were conducted in an individual setting and included a brief reassessment of postural alignment followed by performance of selected exercises. A structured home exercise program was not systematically recorded due to the retrospective nature of this report.

The Canali Orthopedic Brace was prescribed as a complementary component of the conservative intervention. Unlike traditional rigid braces intended for continuous wear, this device is designed for intermittent use and provides frontal and anterolateral restraint without continuous rigid corrective forces [15]. The brace was worn during supervised sessions and for limited daily periods (up to approximately 3 h/day), according to its intended function as a neuromotor facilitation tool rather than a passive corrective orthosis. Its adjustable tension system allows progressive modulation during use, supporting dynamic interaction between the orthosis and the patient’s postural responses.

The overall exercise progression was designed to address postural stability, rotational control, and frontal plane asymmetry in a stepwise manner. The initial phase focused on sagittal plane stabilization through activation and coordination of key stabilization targets, including the abdominal wall, hip extensors (hamstrings), and scapulohumeral stabilizers, to provide a stable proximal reference during trunk control. After sagittal stabilization, rotational control exercises were introduced to reduce asymmetric transverse plane dominance and improve controlled trunk alignment. In the final phase, frontal plane exercises were implemented to reduce lateral asymmetry and rebalance loading patterns, and were introduced only after adequate sagittal and rotational control had been achieved to minimize compensatory strategies. This sequential approach reflects a motor control-based rationale aimed at progressively reducing asymmetrical loading without imposing continuous passive correction.

## 3. Results

A 15-year-old and a 16-year-old patient were referred for postural and radiological evaluation due to a diagnosis of adolescent idiopathic scoliosis. The patients did not report acute pain and had no history of spinal surgery, neurological disorders, or systemic musculoskeletal diseases.

### 3.1. Case 1

**Figure 1:**
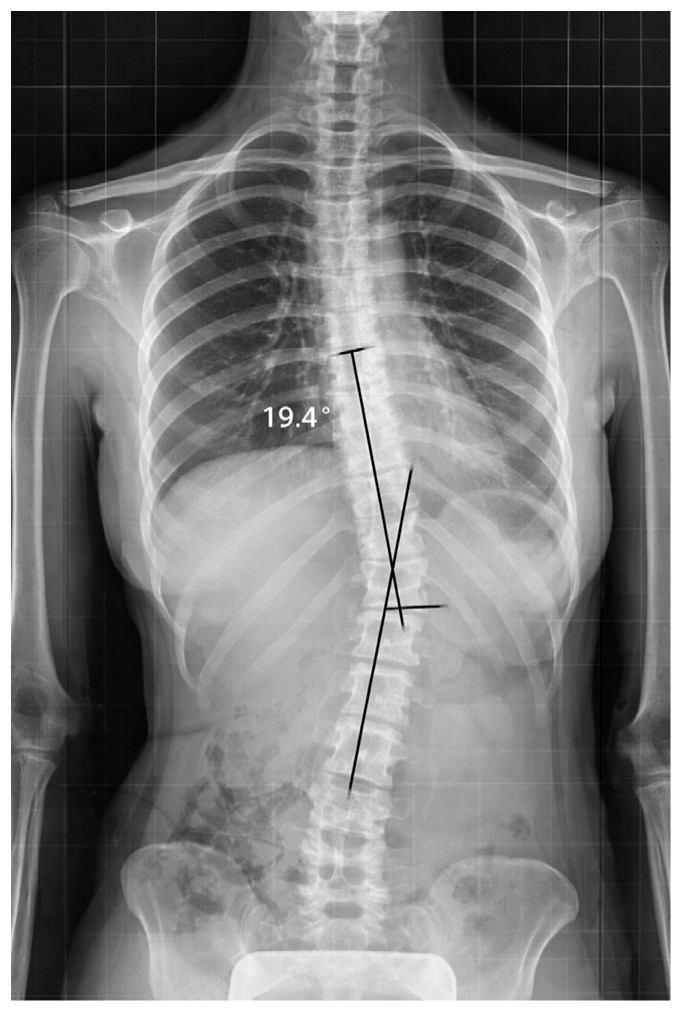
Case 1: Basic standing anteroposterior spinal X-ray showing left-convex thoracolumbar scoliosis (Cobb angle 19.4°). Black lines indicate Cobb angle measurement performed by the radiologist between the upper and lower end vertebrae of the curve.

Standing spinal radiographs revealed left-convex thoracolumbar rotoscoliosis with the curved apex located between T12 and L1. The initial Cobb angle was 19.4°, consistent with a mild-to-moderate scoliotic deformity. Vertebral rotation was present, with a derotation angle ratio (DAR) of 1.9 (Figure 1).

Radiological evaluation also showed an asymmetry of pelvic alignment, with a discrepancy in height of the iliac crest of about 7.1 mm; a rectification of the thoracolumbar sagittal profile; and a preservation of cervical lordosis.

Skeletal maturity was assessed as Risser grade 4, indicating an advanced stage of skeletal development and a lower likelihood of spontaneous curve correction.

Overall, the findings were consistent with thoracolumbar AIS in an advanced stage of skeletal maturity.

### 3.2. Case 2

**Figure 2:**
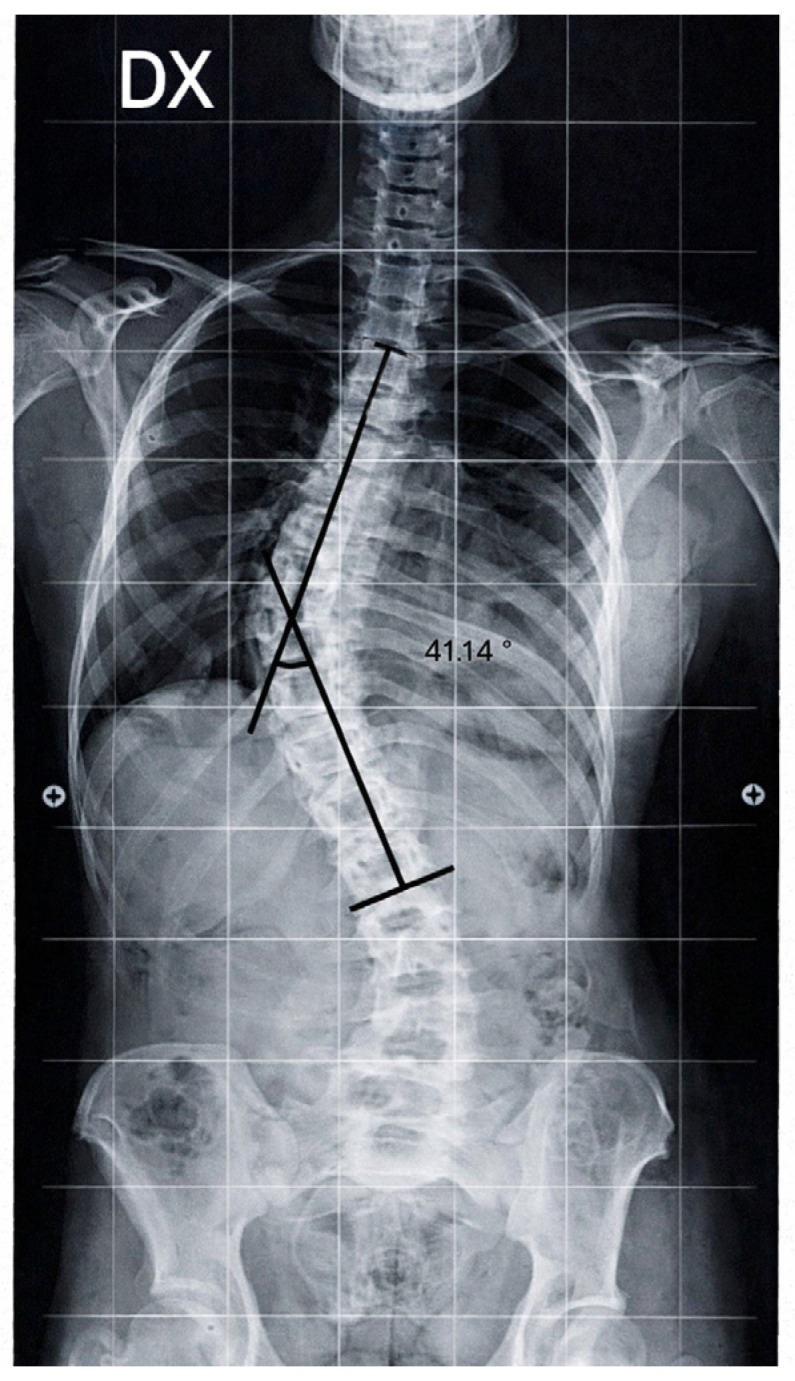
Case 2: Basic standing anteroposterior spinal X-ray showing right-convex thoracolumbar scoliosis (Cobb angle 41.14°). Black lines indicate Cobb angle measurement performed by the radiologist between the upper and lower end vertebrae of the curve.

Standing spinal radiographs revealed right-convex thoracolumbar rotoscoliosis with the curved apex located between T10. The initial Cobb angle was 41.14°, consistent with moderate-to-severe scoliosis. Vertebral rotation was present, with a derotation angle ratio (DAR) of 3.7 (Figure 2).

Radiological evaluation also highlighted an asymmetry of pelvic alignment and vertebral straightening, with loss of the physiological curves of dorsal kyphosis and lumbar lordosis. Skeletal maturity was assessed as Risser grade 4, indicating an advanced stage of skeletal development and a lower likelihood of spontaneous curve correction.

Overall, the findings were consistent with thoracolumbar AIS in an advanced stage of skeletal maturity.

### 3.3. Follow-Up Radiological Outcomes

**Figure 3:**
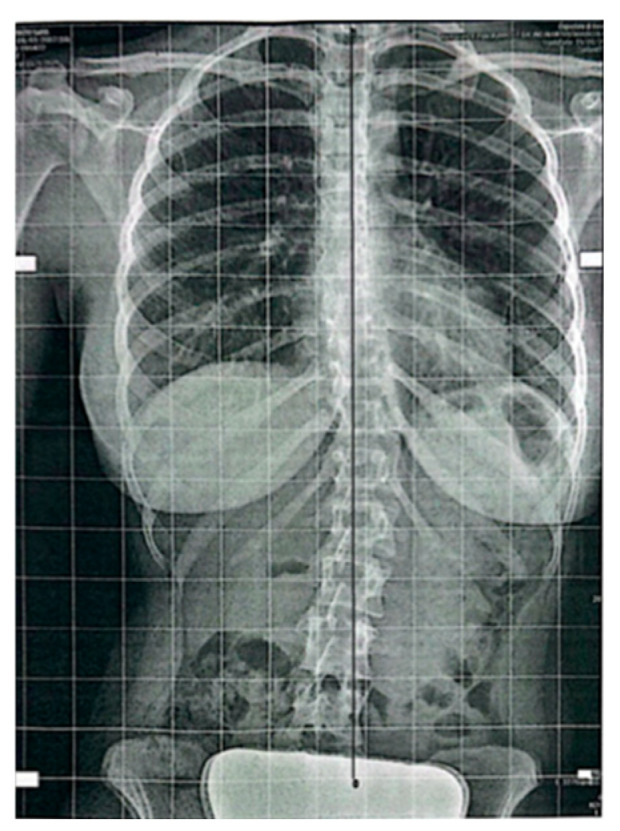
Case 1: Follow-up standing anteroposterior spinal X-ray showing a marked reduction in thoracolumbar curve (Cobb angle 4.1°).

**Figure 4:**
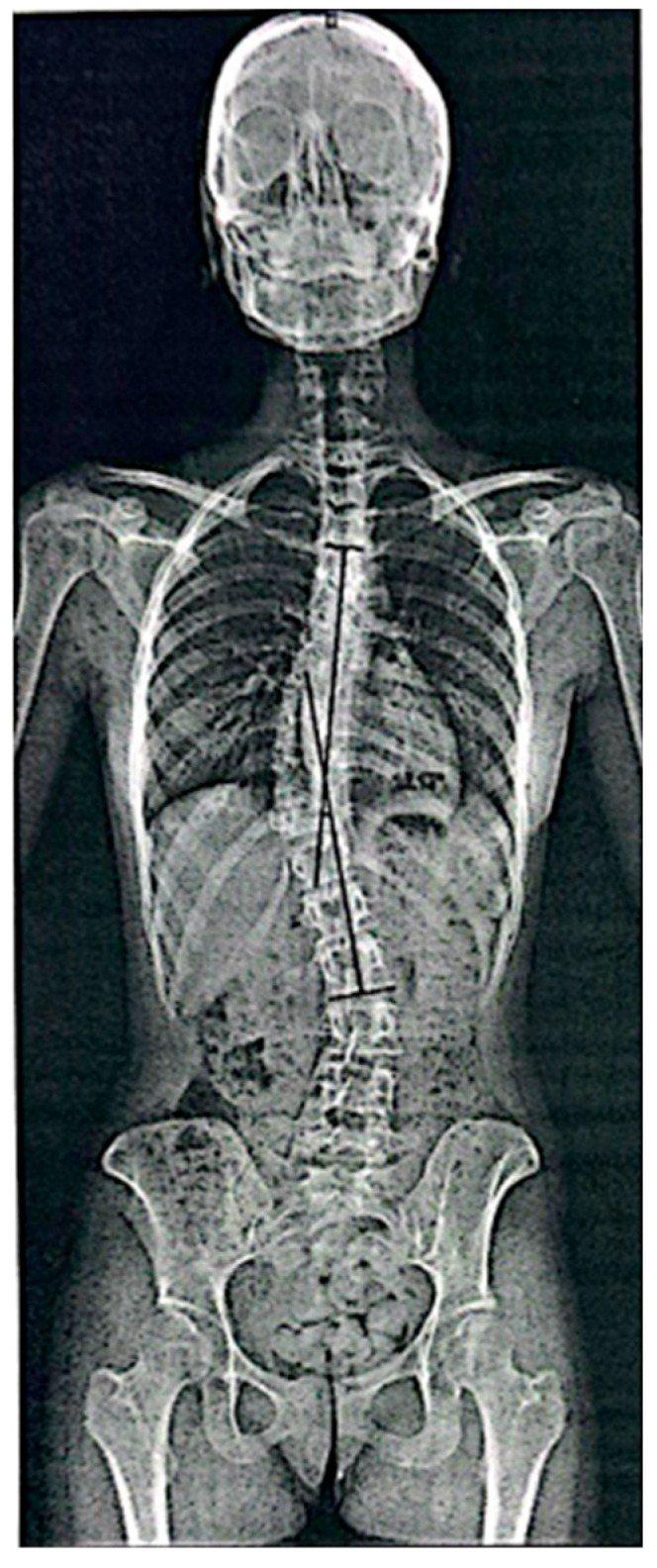
Case 2: Follow-up standing anteroposterior spinal X-ray showing a marked reduction in thoracolumbar curve (Cobb angle 15.17°).

Follow-up standing radiographs showed a clear reduction in coronal plane deviation in both cases. In Case 1, the thoracolumbar Cobb angle decreased from 19.4° at baseline to 4.1° (Figure 3) at follow-up, with a parallel reduction in rotational parameters (DAR from 1.9 to 0.4) and reduced pelvic asymmetry. In Case 2, the Cobb angle decreased from 41.14° to 15.17° (Figure 4), with the DAR decreasing from 3.7 to 1.36 (Table 1). No worsening of sagittal alignment or development of new compensatory curves was observed on follow-up imaging. Radiological measurements were performed by an independent radiologist, and the anonymized radiological report is provided as Supplementary Material S1.

## 4. Outcomes

Radiological outcomes were assessed using standing anteroposterior (AP) and lateral spinal radiographs obtained at baseline and at follow-up. The primary imaging outcomes were Cobb angle measurement and radiological rotational assessment (reported as the derotation angle ratio, DAR), together with pelvic alignment evaluation.

### 4.1. Radiographic Measurements

Cobb angles were measured on standing AP radiographs using the standard end vertebra method [16,17,18]. All radiological measurements reported in this manuscript were performed by an independent radiologist as part of routine clinical practice.

Observers were not blinded to the timepoint due to the retrospective nature of this report. Formal intra- and inter-observer reliability testing was not performed and is acknowledged as a limitation. The original anonymized radiological report and imaging are provided as Supplementary Material S1.

### 4.2. Case 1

At baseline, Case 1 showed a left-convex thoracolumbar curve with an apex at T12–L1. The Cobb angle was 19.4°, and vertebral rotation was documented with a DAR of 1.9. Pelvic asymmetry was also present.

At follow-up, radiographs demonstrated a marked reduction in coronal plane deviation. The Cobb angle decreased to 4.1°, with a parallel reduction in rotational parameters (DAR 0.4). Pelvic asymmetry was reduced compared with baseline, and no worsening of sagittal alignment was observed.

### 4.3. Case 2

At baseline, Case 2 presented with a right-convex thoracolumbar curve with an apex at T10. The Cobb angle was 41.14°, with a DAR of 3.7, consistent with a moderate-to-severe curve and relevant rotational involvement. Pelvic asymmetry was also documented.

At follow-up, radiological evaluation showed a clinically notable reduction in curve magnitude and rotational parameters. The Cobb angle decreased to 15.17°, and the DAR decreased to 1.36. Pelvic asymmetry was reduced compared with baseline, with no evidence of new compensatory curves or sagittal profile deterioration.

### 4.4. Safety

No adverse events were reported during supervised sessions, and no complications were documented in the available clinical notes. However, adverse events were not systematically assessed using a predefined reporting tool.

## 5. Discussion

This retrospective case series reports radiological improvements in two adolescents with thoracolumbar AIS (Risser grade 4) following an integrated conservative program combining supervised CPM-based exercises and intermittent use of a non-rigid orthopedic brace. Although the present design does not allow for causal inference, the magnitude and consistency of the observed changes across both cases support the clinical plausibility of a motor control-oriented strategy targeting postural alignment in multiple planes.

Current evidence-based conservative management of AIS primarily aims to prevent curve progression during growth through prolonged use of rigid or semi-rigid braces, often combined with exercise-based interventions. International recommendations support the use of bracing and physiotherapeutic scoliosis-specific exercises (PSSEs) as key non-surgical options for selected patients, with the goal of promoting active self-correction and three-dimensional postural control. The present approach shares the principle of individualized, active postural training, but differs in the proposed stepwise exercise sequencing and in the intermittent use of a non-rigid brace intended to facilitate postural awareness rather than provide continuous passive correction.

A notable aspect of these cases is the advanced skeletal maturity (Risser grade 4), a stage at which substantial spontaneous curve regression is generally considered less likely. Nevertheless, alternative explanations must be considered. These include natural history, postural adaptation, variability in patient positioning during radiographic acquisition, and radiographic measurement variability. Cobb angle measurements are known to show intra- and inter-observer variability within a few degrees, and formal reliability testing was not feasible in this retrospective report. Importantly, the magnitude of change observed in both cases exceeded typical measurement error, although this observation should be interpreted as hypothesis-generating.

In both cases, improvements were observed not only in coronal plane deviation but also in rotational parameters (DAR) and pelvic alignment, with no worsening of sagittal profile. While these findings may support the interpretation of a clinically meaningful postural change rather than random fluctuation, the absence of standardized clinical outcomes prevents any conclusion regarding symptoms, function, or quality of life. Future prospective studies should include validated patient-reported outcomes (e.g., SRS-22), pain assessment, and functional measures alongside standardized radiological follow-up [19,20].

The present report has additional limitations. The intervention dose differed substantially between cases, and adherence to any home-based component was not systematically documented. Moreover, adverse events were not collected using a predefined reporting tool. These aspects limit comparability and generalizability. Despite these limitations, the cases provide a rationale for further investigation of integrated conservative strategies combining supervised motor learning-based exercise and intermittent orthotic facilitation. Future research should adopt prospective designs, include standardized PSSE comparisons when feasible, and assess the durability of radiological and clinical outcomes over a longer follow-up.

## 6. Conclusions

This retrospective case series describes radiological improvements in two adolescents with thoracolumbar AIS treated with a conservative integrated approach combining supervised CPM-based postural exercises and intermittent use of a non-rigid orthopedic brace. Given the small sample size, retrospective design, heterogeneity in treatment dose, and lack of standardized clinical outcomes and measurement reliability testing, these findings should be interpreted with caution and cannot establish efficacy. However, the observed reductions in Cobb angle and rotational parameters support the plausibility of further research on motor control-oriented rehabilitation strategies combined with intermittent orthotic facilitation. Prospective controlled studies integrating radiological and patient-centered outcomes are warranted.

## Figures and Tables

**Table 1:** Comparison of Cobb angle and DAR before and after treatment.

Case	Cobb Baseline—Follow Up	DAR Baseline—Follow Up
1	19.4–4.1°	1.9–0.4
2	41.14–15.17°	3.7–1.36

## Data Availability

The radiological images supporting the main findings are included in the manuscript and Supplementary Materials. Additional clinical and imaging-derived data are not publicly available due to ethical and privacy restrictions related to sensitive clinical and radiological data from minor patients. Anonymized data are available from the corresponding author upon reasonable request for scientific purposes.

## Supplementary Materials

The following supporting information can be downloaded at: https://www.mdpi.com/article/10.3390/diagnostics16040514/s1, Supplementary Materials S1: Anonymized radiological report (in Italian) provided by the attending radiologist, including descriptive assessment of the scoliotic curve and follow-up evaluation.

## Abbreviations

The following abbreviations are used in this manuscript:

CPM	Canali Postural Method
