# Supplementary material for: Radiological Improvement of Adolescent Idiopathic Scoliosis Following an Integrated Postural Reprogramming Approach: A Retrospective Case Series
Source: Diagnostics (Basel). 2026 Feb 9;16(4):514. doi: 10.3390/diagnostics16040514 (PMC12939006; doi:10.3390/diagnostics16040514)
Supplement: Supplementary file 1 [file diagnostics-16-00514-s001.zip › diagnostics-4096445-supplementary.pdf]

Supplementary Material S1
Original Radiological Report and Imaging (Italian)

This supplementary file contains the original anonymized radiological report and corresponding spinal radiographs referenced in the manuscript.

PAZIENTE NUMERO 1

Paziente affetta da rotoscoliosi toracolombare sinistroconvessa con fulcro al passaggio tra i somi vertebrali di T12-L1.

Si associa perdita delle fisiologiche curve sul piano sagittale di cifosi dorsale e lordosi lombare, laddove i tratti di colonna paiono rettilineizzati. Sostanzialmente conservata la fisiologica lordosi cervicale.

La curva risulta sbilanciata a sn con associato slivellamento delle creste iliache.

Morfologia dei somi vertebrali conservata. Nella norma l'altezza degli spazi intersomatici

Al controllo a sei mesi si apprezza riduzione dell'ampiezza della curva scoliotica. Ridotto anche lo slivellamento delle creste iliache

Tuttora invariata la rettilineizzazione del rachide toraco-lombare.

SDS

Paziente affetta da rotoscoliosi toracolombare sinistroconvessa con fulcro al passaggio tra i somi vertebrali di T12-L1.

Si associa perdita delle fisiologiche curve sul piano sagittale di cifosi dorsale e lordosi cervicale e lombare, laddove i tratti di colonna paiono rettilineizzati.La curva risulta sbilanciata a sn con associato slivellamento delle creste iliache di 7,1 mm circa

Morfologia dei somi vertebrali conservata. Nella norma l'altezza degli spazi intersomatici.

Risser grado 4

DAR 1,9

Al controllo dopo trattamento si apprezza riduzione dell' ampiezza della curva scoliotica, attualmente con angolo di Cobb da 19,4° a 4,1° (DAR 0,4)

Dopo trattamento si apprezza anche dello slivellamento delle creste iliache mentre pare sovrapponibile la rettilineizzazione del rachide.

Dott. Francesco Tucci
Medico Chirurgo
Specialista in Radiodiagnostica
TCCFNC94B25A662K

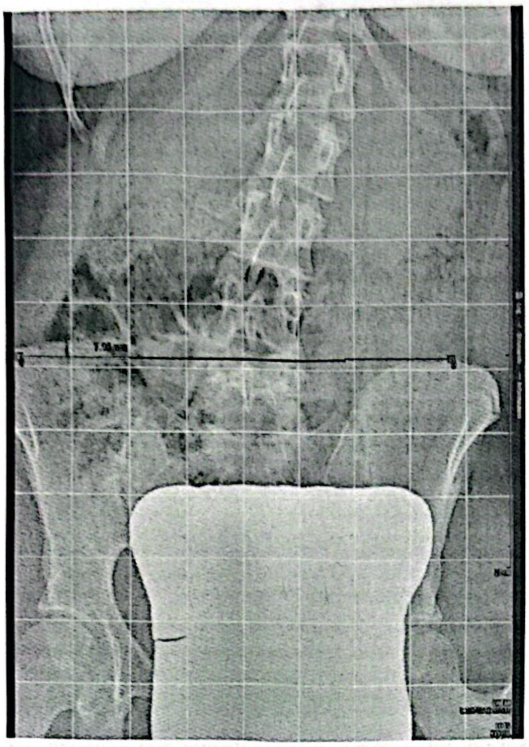

Dott. Francesco Tucci
Medico Chirurgo
Specialista in Radiodiagnostica
TCCFNC94B25A662K

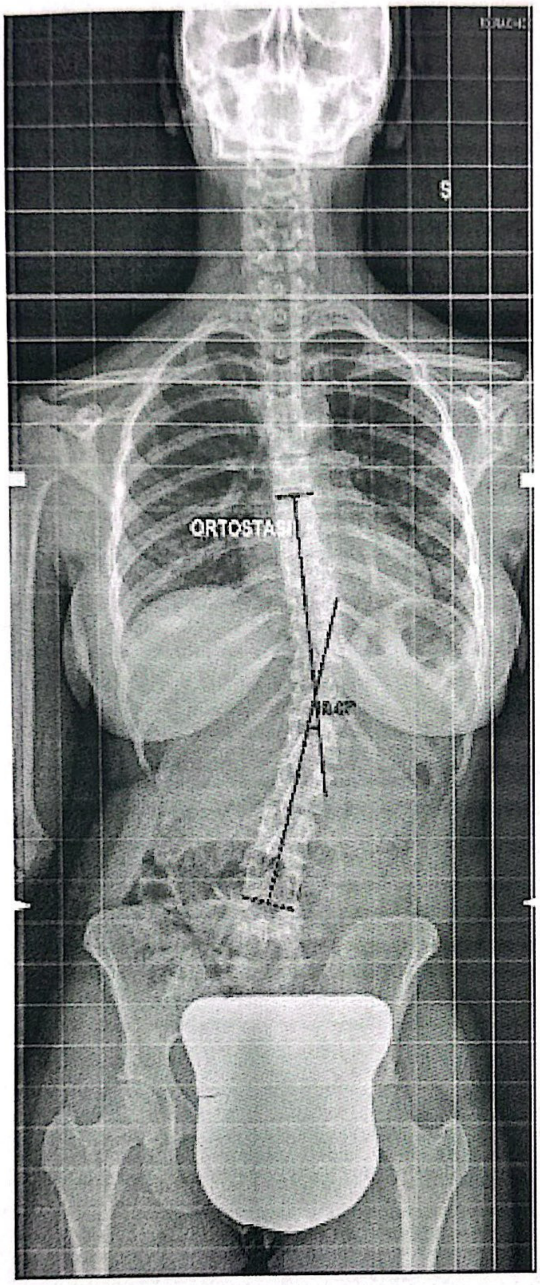

Dott. Francesco Tucci
Medico Chirurgo
Specialista in Radiodiagnostica
TCCFNC94B25A662K

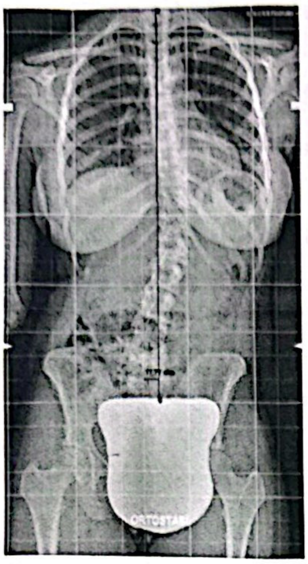

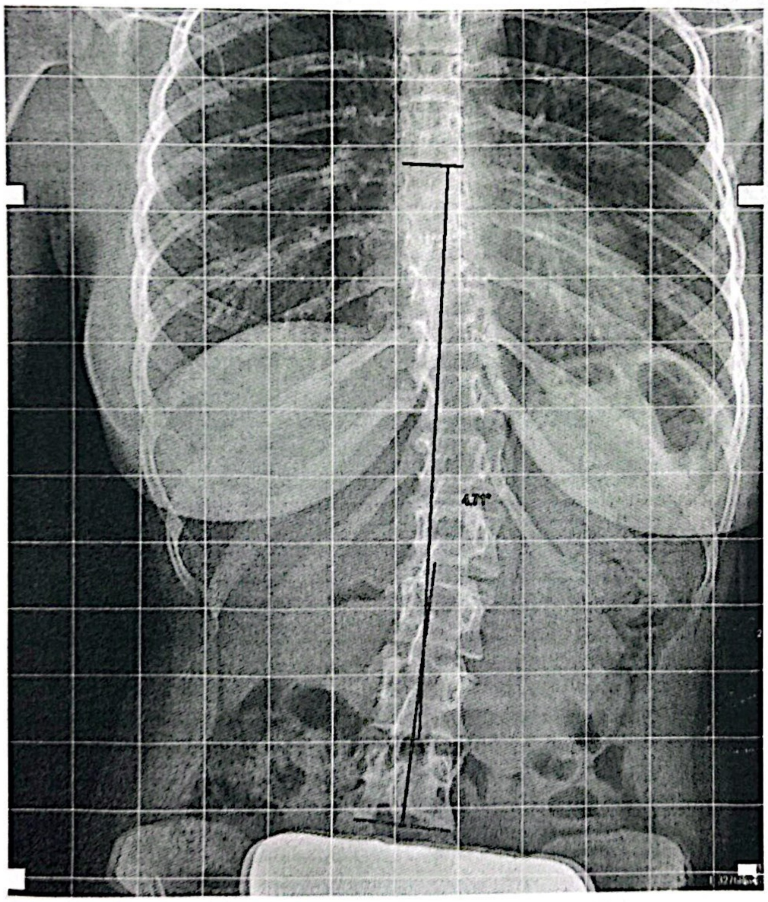

Dott. Francesco Tucci
Medico Chirurgo
Specialista in Radiodiagnostica
TCCFNC94B25A662K

Case 2

ML

Paziente affetta da scoliosi toracolombare destroconvessa con fulcro al passaggio tra i somi vertebrali di T10, con associata curva di compenso a livello lombare.

Si associa perdita delle fisiologiche curve sul piano sagittale di cifosi dorsale e lordosi cervicale e lombare, laddove i tratti di colonna paiono rettilineizzati.

La curva risulta sbilanciata a dx con associato slivellamento delle creste iliache di 4mm circa

Morfologia dei somi vertebrali conservata. Nella norma l'altezza degli spazi intersomatici.

Risser grado 4

DAR 3,7

Dott. Francesco Tucci
Medico Chirurgo
Specialista in Radiodiagnostica
TCCFNC94B25A662K

Al controllo dopo trattamento si apprezza netta riduzione dell' ampiezza della curva scoliotica, attualmente con angolo di Cobb da 41,14° a 15,17 °con riduzione dello slivellamento delle creste iliache e miglioramento del bilanciamento sul piano coronale (DAR 1,36)

Minima riduzione a carico dello slivellamento delle creste iliache

Tuttora invariata la rettilineizzazione del rachide toraco-lombare.

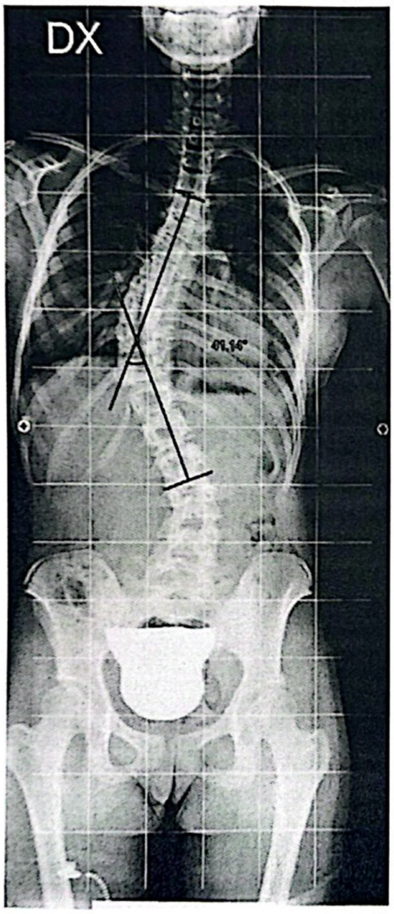

Dott. Francesco Tucci
Medico Chirurgo
Specialista in Radiodiagnostica
TCCFNC94B25A662K

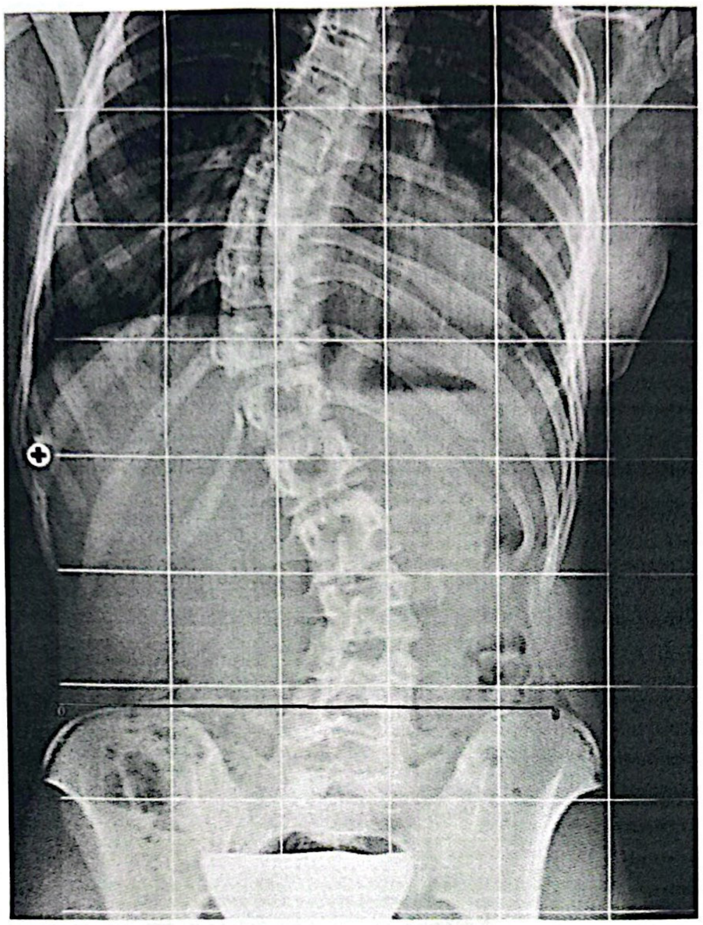

Dott. Francesco Tucci
Medico Chirurgo
Specialista in Radiodiagnostica
TCCFNC84B25A662K

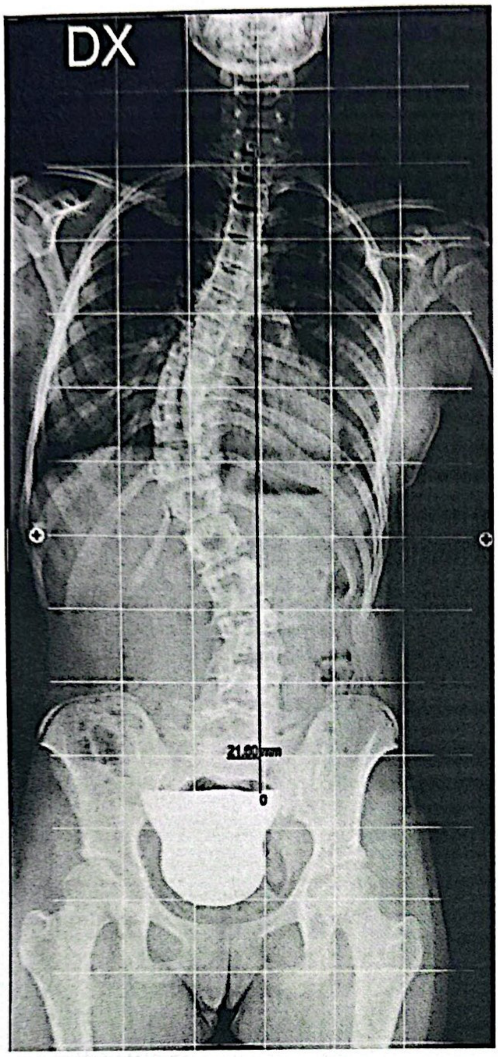

Dott. Francesco Tucci
Medico Chirurgo
Specialista in Radiodiagnostica
TCCFNC94B25A662K

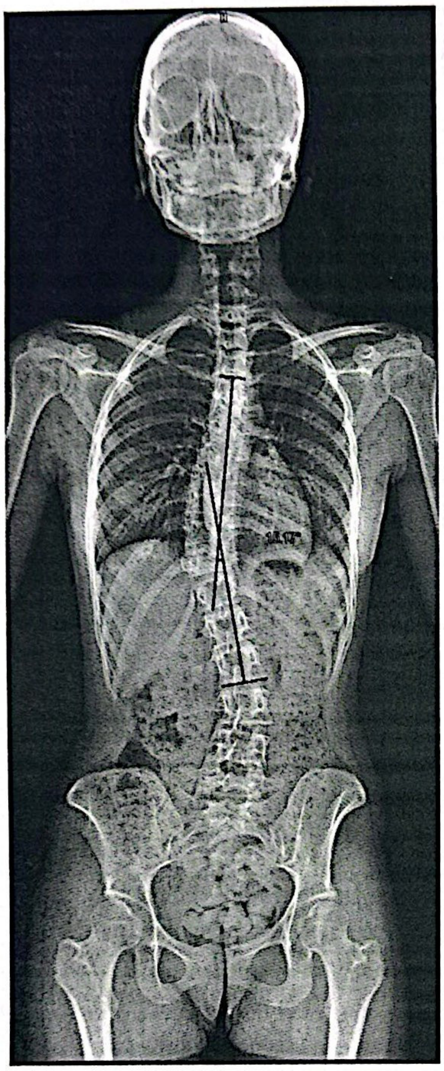

Dott. Francesco Tucci
Medico Chirurgo
Specialista in Radiodiagnostica
TCCFNC94B25A662K

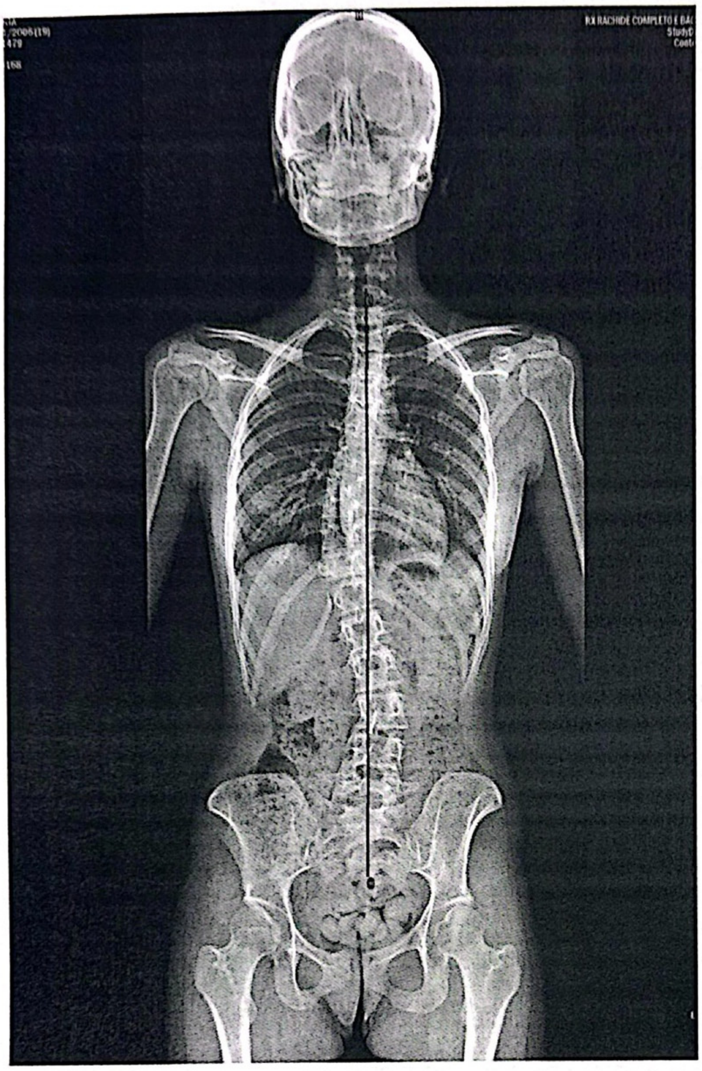

Dott. Francesco Tucci
Medico Chirurgo
Specialista in Radiodiagnostica
TCCFNC94B25A662K

REFERTO STRUTTURATO – VALUTAZIONE SISTEMATICA DELLE RADIOGRAFIE

Analisi di immagini RX rachide in toto acquisite in modo da comprendere nei volumi dal capo sino alle articolazioni coxofemorali.

I referti sono stati redatti seguendo il seguente schema, in modo da dare organicità alla valutazione delle immagini pre e post-trattamento, nonché omogeneità nei criteri presi in analisi per i Pazienti attualmente arruolati nello studio, garantendola anche per i Pazienti futuri.

-FULCRO DELLA SCOLIOSI

-ANGOLO DI COBB indicato in gradi

-VALUTAZIONE DI UNA EVENTUALE CURVA MINORE compensatoria

-ALTERAZIONI DELLE CURVE SUL PIANO SAGITTALE

-BILANCIAMENTO DELLA CURVA RISPETTO ALLA LINEA MEDIANA calcolato misurando la distanza orizzontale della linea a piombo da C7 rispetto alla linea mediana sacrale

-SLIVELLAMENTO CRESTE ILIACHE

-DAR (deformity angular ratio) che rappresenta un modo indiretto per misurare la severità di una curva scoliotica, dal momento che rappresenta il rapporto tra la massima deformità espressa in angoli di Cobb divisa per il numero di livelli che vengono compresi in una curva. Ad un DAR pù elevato si associa una maggiore severità della curva.

Dott. Francesco Tucci
Medico Chirurgo
Specialista in Radiodiagnostica
TCCFNC84B25A662K
